# Agreement between Two Methods of Dietary Data Collection in Male Adolescent Academy-Level Soccer Players

**DOI:** 10.3390/nu7075262

**Published:** 2015-07-17

**Authors:** Marc A. Briggs, Penny L. S. Rumbold, Emma Cockburn, Mark Russell, Emma J. Stevenson

**Affiliations:** 1 Department of Sport, Exercise and Rehabilitation, Faculty of Health and Life Sciences, Northumbria University, Newcastle upon Tyne NE1 8ST, UK; E-Mails: penny.rumbold@northumbria.ac.uk (P.L.S.R.); mark.russell@northumbria.ac.uk (M.R.); e.stevenson@northumbria.ac.uk (E.J.S.); 2 London Sport Institute, Middlesex University, London NW4 4BT, UK; E-Mail: e.cockburn@mdx.ac.uk

**Keywords:** energy intake assessment, food diaries, 24-h recall, football, adolescent, male

## Abstract

Collecting accurate and reliable nutritional data from adolescent populations is challenging, with current methods providing significant under-reporting. Therefore, the aim of the study was to determine the accuracy of a combined dietary data collection method (self-reported weighed food diary, supplemented with a 24-h recall) when compared to researcher observed energy intake in male adolescent soccer players. Twelve Academy players from an English Football League club participated in the study. Players attended a 12 h period in the laboratory (08:00 h–20:00 h), during which food and drink items were available and were consumed *ad libitum.* Food was also provided to consume at home between 20:00 h and 08:00 h the following morning under free-living conditions. To calculate the participant reported energy intake, food and drink items were weighed and recorded in a food diary by each participant, which was supplemented with information provided through a 24-h recall interview the following morning. Linear regression, limits of agreement (LOA) and typical error (coefficient of variation; CV) were used to quantify agreement between observer and participant reported 24-h energy intake. Difference between methods was assessed using a paired samples *t*-test. Participants systematically under-reported energy intake in comparison to that observed (*p* < 0.01) but the magnitude of this bias was small and consistent (mean bias = −88 kcal·day^−1^, 95% CI for bias = −146 to −29 kcal·day^−1^). For random error, the 95% LOA between methods ranged between −1.11 to 0.37 MJ·day^−1^ (−256 to 88 kcal·day^−1^). The standard error of the estimate was low, with a typical error between measurements of 3.1%. These data suggest that the combined dietary data collection method could be used interchangeably with the gold standard observed food intake technique in the population studied providing that appropriate adjustment is made for the systematic under-reporting common to such methods.

## 1. Introduction

Optimizing nutritional intake is imperative for adolescents to maintain health, growth and maturation [1]. Furthermore, optimal energy intake is required to meet the training and competition demands of highly active adolescents [1,2]. Sufficient energy intake in adolescents engaged in high training volumes may result in improved bone health, aid maturational development and decrease risk of injuries [3]. Accurately understanding the nutritional requirements of adolescents can support such health benefits and fuel the body for optimal performance.

Collecting accurate and reliable nutritional data from adolescent populations is troublesome [4]; for example, quantifying energy intake utilizing self-reported methods within an adolescent population presents challenges, such as under-reporting and a lack of detailed information [5]. Although research has focused on quantifying the energy intake of children [6,7,8,9], female adolescents [6,10,11,12,13] and obese adolescent [10,14] populations, limited data exists for the quantification of energy intake in highly active male adolescents such as academy soccer players who are training and competing up to 20 h per week [15], with daily energy expenditure ranging from 12.5–15.2 MJ·day^−1^ (2986–3631 kcal·day^−1^) [16,17]. Traditional methods of assessing energy intake within normal weight, male adolescent populations, albeit not highly trained, have included estimated and weighed food records and diet histories [6,10,11,18]. Studies have investigated the validation of self-reported energy intake against doubly labelled water (DLW) measurements, demonstrating an underestimation of energy intake and fluid consumption by 18%–27% [6,10,11,18]. Underestimation of energy intake in comparison to total energy expenditure is consistent within the literature, providing detailed explanations for the reporting error [6,10,11,18]. However, further research is required to quantify the actual reporting accuracy of energy intake, which previous studies fail to address [6,10,11,18].

Contextualizing the environment in which the participant is recording energy intake is of equal importance. Previous pediatric research has focused on assessing energy intake, albeit in laboratory conditions, with intake recorded by observers [19,20,21]. However, research representing real-world environments is imperative in understanding habitual energy intake. Recent studies have attempted to incorporate a more representative real-world design [22], acknowledging a compromise between the high internal validity of laboratory-based studies, whilst attempting to provide ecological validity incorporated through more free-living designs [23]. It is important when conducting research with adolescent populations to accommodate a balance between high internal and high ecological validity, to ensure findings can be extrapolated accurately [24].

It has been suggested that introducing a combined method of dietary data collection in the form of self-reported, weighed food diaries and 24-h recall interviews can increase the accuracy of self-reported energy intake measurements in adolescent and child populations [22,25,26]. Rumbold *et al.* [26] investigated the accuracy of this combined method by exploring the agreement between researcher observed and recorded energy intake and self-reported energy intake in female adolescent netballers (14–16 years old). Although this was within a laboratory setting, a range of food and drink items were made available *ad libitum* to represent real-world conditions, as items were based on previous free-living self-reported food diaries. Results indicated a slight bias towards over-reporting of 0.46 MJ·day^−1^ (110 kcal·day^−1^). These findings are in contrast to previous published data of significant under-reporting in adolescent populations [6,10,11,18]. Rumbold *et al.* [26] concluded that on a group level (0.00 to 0.92 MJ·day^−1^, 0 to 220 kcal·day^−1^) the combined dietary data collection method is an effective approach to quantify energy intake in adolescent female netballers. However, caution is required when extrapolating to other population groups, such as highly-trained, male counterparts.

A relatively limited number of studies have focused on nutritional recommendations for the male adolescent soccer player [16,17,27,28,29,30,31,32]. As soccer is perceived to be one of the most popular sports worldwide [33], this highlights the importance of providing research-informed nutritional recommendations based on growth, health, maturation and training status [1,34]. High-level soccer players will generally have higher intake requirements due to the greater energy expenditure from training and competition [34]. This highlights the importance of accurately quantifying energy intake to ensure the energy expenditure demands of training and competition are met. Accurate methods are required for both field-based researchers and highly trained practitioners to provide evidence-based interventions and recommended nutritional practices. Therefore, the aim of the current study is to explore the agreement between researcher observed energy intake and self-reported energy intake in male adolescent high-level soccer players using a combined self-reported, weighed food diary and 24-h recall method.

## 2. Experimental Section

### 2.1. Design

Diet was assessed for each participant over 24 h (12 h spent in the nutrition Laboratory at Northumbria University (08:00 h–20:00 h), followed by 12 h spent at home between 20:00 h and 08:00 h the following morning). Participants were informed that the study was based on a general energy intake analysis, however, were not informed that their ability to record their food and fluid intake was being monitored. The combined method of energy intake comprised of a self-reported weighed food diary with a subsequent 24-h recall. The research observed energy intake utilized a coding system having covertly weighed all available food and drink items.

### 2.2. Participants

Twelve males (age: 13.8 ± 0.6 years; stature: 1.71 ± 0.04 m; body mass: 63.7 ± 5.0 kg; Body Mass Index: 21.9 ± 1.9 kg·m^−2^) were selected for the study. The maturity offset was 2.2 ± 0.4 years from peak height velocity indicating that all of the participants had reached their predicted peak height velocity (positive maturity offset) and thus were of a similar maturation status [35]. All participants were training with a football academy and this included training at least three times per week in addition to a match day. This equated to 7.5 h of moderate to high-intensity intermittent activity per week. To determine if the participants were restrained or unrestrained eaters, the Dutch Eating Behavior Questionnaire [36] was administered. All participants were classified as unrestrained eaters, with the mean (±standard deviation (SD)) dietary restraint score (2.3 ± 0.3) falling into the average range for high school males [36]. The study was approved by the Faculty of Health and Life Sciences Research Ethics Committee at Northumbria University (approval number RE09-11-11307, 11 January 2012). Written informed consent was gained from the participants and their parents or guardians prior to data collection.

### 2.3. Protocol

Prior to the study, a workshop was conducted, during which the participants were provided with a detailed explanation and demonstration of the food weighing and recording process. The workshop provided the participants with the opportunity to practice this technique and the 24-h recall interview in the presence of a researcher, as recommended by Livingstone *et al.* [6].

All participants attended the Northumbria University Nutrition Laboratory between 08:00 h and 20:00 h. During the visit, participants were kept occupied with a range of inactive tasks such as reading and homework. Participants were provided with breakfast, lunch and dinner, as well as snacks *ad libitum* during the day. To replicate a real-world environment, food and drink items provided were based on a previously administered food preference questionnaire. Details of all foods provided are outlined in Table 1. The research team were responsible for preparing and covertly weighing all available food and drink items to the nearest gram or millilitre and producing a numerical code for each item. Participants were informed all food and drink items were available *ad libitum* to replicate real-world conditions, although instructions were provided to enable participants to weigh (Sartorius TE6100, Goettingen, Germany) and record all of the food and drink consumed in a food diary provided. To enhance energy intake accuracy, all leftover food was weighed and recorded by the participant and also covertly weighed and disposed of by the researcher.

**Table 1:** Food and drink items made available for the subjects during the study.

Meal	Food and Drink Items Available
Breakfast	Kellogg’s Frosties, Coco pops, Cornflakes, Honey loops, Coco “Choc n Roll”, Rice Krispies Multi-Grain, Rice Krispies, Weetabix, semi-skimmed or whole milk
Lunch	Ham Sandwich white/brown bread with/without butter, Chicken Sandwich white/brown bread with/without butter
Dinner	Jacket potato (with/without beans, cheese), Tomato pasta (with/without cheese), Chicken breast (with/without potatoes, carrots, tomato sauce)
Secondary Items	Orange cordial (no added sugar), water, pure apple juice, pure orange juice, fruit (bananas, apples, clementines), yoghurts, cereal bars, crisps, confectionary

For the period between 20:00 h and 08:00 h the following morning, whilst at home, participants were required to only consume items taken from the laboratory and follow the same weighing process as in the laboratory. Therefore, secondary food and drink items, plus cereal and milk cartons, were available to take home for consumption (see Table 1). However, no participants opted to take any additional items home; therefore no further consumption was recorded between 20:00 h–08:00 h. The following morning individual face-to-face 24-h recall interviews were conducted using the two-pass method [26,37]. This method firstly reviews the main foods and beverages consumed within the 24-h period, whilst secondly prompting participants for more information such as condiments, brand names, how the foods were prepared and cooked and portion sizes if not provided in the first pass.

Twenty-four-hour energy intake (12 h in the laboratory and 12 h at home; which will be referred to as “observed” intake for the remainder of this paper) was determined for each participant by the researcher using the covert numerical coding system (as explained above). Twenty-four-hour participant self-reported food intake was determined using the information provided in the self-reported weighed food diaries, which was confirmed and supplemented with any additional information the participants provided during the 24-h recall interviews. This combined dietary data collection method has previously been used by Rumbold *et al.* [26] in a recreationally active female population of a similar age.

### 2.4. Estimation of Energy Intake

To calculate the observed and participant reported 24-h energy intake (MJ·day^−1^) the nutritional content of all food and drink items were obtained using the food packaging and analyzed in Microsoft Excel. When food portions were not identified by the participants, amounts were substituted using a portion size recorded in the individual’s food diary which corresponded to an identical food or drink item.

### 2.5. Analysis

All data are presented as mean ± SD. The agreement between estimates of energy intake (MJ·day^−1^) reported by the participants (self-reported, weighed food diaries and 24-h recall interviews) and observed energy intake by the researcher was assessed using a range of statistics. Limits of agreement (LOA) using the Bland and Altman [38] method was used to assess the relative bias (mean difference) and random error (1.96 SD of the difference) between methods, as recommended by Livingstone *et al.* [6]. 95% Confidence intervals (CI) for the bias and paired samples *t*-tests were used to test for significant differences between methods. Random error was further assessed using typical error of the estimate as a coefficient of variation (CV), and linear regression [39]. Statistical significance was assumed at *p* < 0.05.

## 3. Results

Participants self-reported energy intake (11.87 ± 2.01 MJ·day^−1^, 2835 ± 480 kcal·day^−1^) was significantly under-reported in comparison to observed energy intake (12.23 ± 2.12 MJ·day^−1^, 2921 ± 506 kcal·day^−1^) with a mean bias of −0.37 MJ·day^−1^, −88 kcal·day^−1^ (95% CI for bias = −0.61 to −0.12 MJ·day^−1^; −146 to −29 kcal·day^−1^) (*t*(11) 3.291, *p* = 0.007). The combined approach of self-reported, weighed food diary and 24-h recall therefore had a 3.0% bias towards under-reporting of energy intake when male adolescent, high-level soccer players are asked to record their food and fluid intake, though the 95% CI for this bias was narrow with a range of 1.0% to 5.0%.

**Figure 1:**
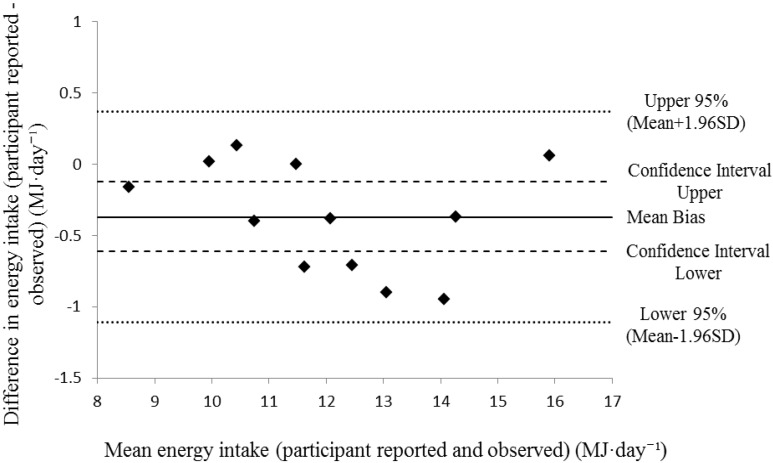
Individual differences in energy intake (participant reported energy intake − observed energy intake) plotted against the mean of the measurements for energy intake.

**Figure 2:**
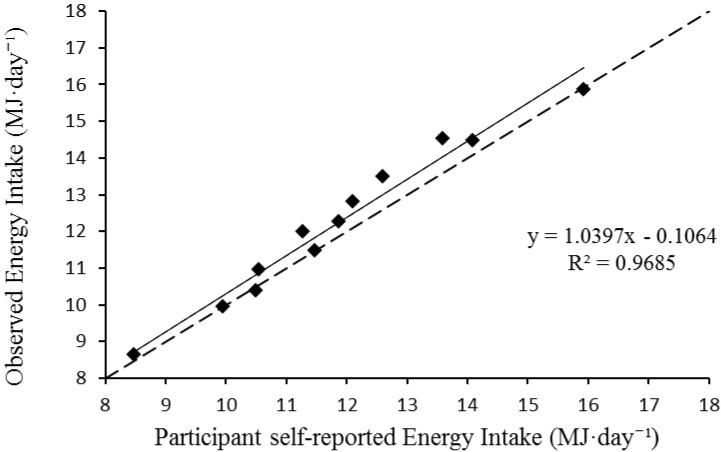
Linear regression scatter plot. The dashed line represents the line of equality. The solid line denotes the regression line.

For random error, the 95% LOA between methods ranged between −1.11 to 0.37 MJ·day^−1^ (−256 to 88 kcal·day^−1^, Figure 1). The standard error of the estimate was low, with a typical error between measurements of 3.1%. The results of the linear regression analysis (and associated calibration equation) are presented in Figure 2. Collectively these data demonstrate a low degree of random error between self-reported energy intake and researcher observed methods. A visual inspection of the distribution of data in Figure 1 and Figure 2 show no indication of hetroscedasticity.

## 4. Discussion

The aim of the current study was to explore the agreement between researcher observed energy intake and self-reported energy intake in male adolescent high-level soccer players using a combined method (self-reported weighed food diary, supplemented with a 24-h recall). The findings demonstrate the variability between methods was low (typical error of the estimate = 3.1%) and although a statistically significant under-reporting was observed with the combined self-report method, the magnitude of this bias was both small (−0.37 MJ·day^−1^; −88 kcal·day^−1^) and consistent (95% CI for bias = −0.61 to −0.12 MJ·day^−1^; −146 to −29 kcal·day^−1^). Consequently, with an appropriate adjustment for under-reporting, the combined self-report method could be used as an alternative to the researcher observed method to quantify energy intake in male adolescent high-level soccer players. Furthermore, it could be a valuable tool to adopt when studying such measures in a free-living environment.

Participant mean self-reported energy intake (11.87 ± 2.01 MJ·day^−1^; 2835 ± 480 kcal·day^−1^) produced a significant bias of −0.37 MJ·day^−1^ (−88 kcal·day^−1^) toward under-reporting when compared to mean observed energy intake (12.23 ± 2.12 MJ·day^−1^; 2921 ± 506 kcal·day^−1^). However, when analyzing agreement between two methods it is important to question whether the differences are meaningful. Bland and Altman [40] suggest that it is not the statistical difference that matters, but the magnitude. While the difference between methods was significantly different, the magnitude of the difference was low, as evidenced by the narrow confidence interval for bias (−0.61 to −0.12 MJ·day^−1^; −146 to −29 kcal·day^−1^). Wang *et al.* [41] proposed a difference in energy intake of 110–165 kcal·day^−1^ to be clinically meaningful in a weight loss context; higher than the mean bias identified in the current study (−88 kcal·day^−1^), suggesting the under-reporting would not likely impact on energy balance. Importantly for future studies, the degree of random error between methods was also low, with a typical error of the estimate of 3.1%, with 95% LOA ranging from −1.11 to 0.37 MJ·day^−1^ (−256 to 88 kcal·day^−1^). Therefore it is suggested that future studies may adopt the self-report method for determining energy intake within this population, with a small adjustment for the likely small, but significant, under-reporting. If future study findings required an adjustment, this would be achieved using the calibration equation provided by the linear regression analysis in Figure 2; *y* = 1.0397*x* − 0.1064 where *y* = researcher observed energy intake and *x* = participants self-reported energy intake. To illustrate, for the average self-report energy intake measured in this study (11.87 MJ·day^−1^) would be adjusted to 12.23 MJ·day^−1^ to account for the under-reporting using the calibration equation. Using this method will allow researchers and practitioners to accurately investigate energy intake in free-living, field-based environments and as such could incorporate more accurate nutritional interventions to optimize performance.

In an attempt to quantify a range in which LOA are deemed acceptable as an effective method, the current study’s results were compare to that of Livingstone *et al.* [6] and Rumbold *et al.* [26] which also utilized the Bland and Altman [38] method of analysis to assess validity of energy intake. The current study findings demonstrate narrower ranges of both 95% LOA and 95% CI to these previous studies.

Although no previous research has attempted to quantify the accuracy of energy intake within the population of adolescent high-level soccer players, the finding of significant under-reporting is in agreement with the majority of previously published studies investigating energy intake methods in adolescent males [6,10,11,18]. Despite studies adopting differing methods of collecting dietary energy intake, results present unequivocal evidence of significant under-reporting when validated against DLW; −20%; −22%; −18% and −22% respectively [6,10,11,18]. Whilst the current study is in agreement with previous studies with regards to identifying a significant under-reporting of energy intake, the considerably lower −3% error highlights a substantial improvement when a combined dietary data collection method is adopted.

The low level of bias and close agreement demonstrated in the current study can be explained by the participants’ high level of engagement. Livingstone *et al.* [6] discussed the effect of age on validity of energy intake, suggesting the magnitude of under-reporting increases as children enter adolescence. However, educational workshops were conducted with the participants providing a detailed explanation and demonstration of the food weighing and recording process, increasing participant’s engagement and confidence with the collection method. Furthermore, athletes have been found to display traits of higher inherent motivation levels [42,43], which may account for the higher level of compliance and engagement in the combined collection method within the current population sample, due to their willingness to develop, learn and impress academy staff. Participants were also administered the Dutch Eating Behaviour Questionnaire [36] prior to data collection. All twelve subjects were classified as unrestrained eaters, with the mean (±SD) dietary restraint score (2.28 ± 0.3) falling into the average range for high school males [36]. Higher levels of dietary restraint are more likely to coincide with under-reporting, as this was not evident, as well as all participants recording a healthy body mass index (21.9 ± 1.9 kg/m^2^); it is likely that this also contributed to the small bias of the self-reported energy intake.

One possible reason to account for the small under-reporting evident in the present study could be the nature of how athletes consume nutrients. Frequent snacking is extremely common amongst the athlete population to accomplish the high energy intake requirements of high-level sport [44], with as many as nine occasions of snacking demonstrated over a single day period [45]. Therefore the eating pattern of adolescent athletes may be more complex due to increased frequency of recording, which can subsequently increase burden and effect compliance levels [25] as well as increase the difficulty of remembering large amounts of food items during 24-h recall. The mean observed energy intake in the current study was 12.23 ± 2.12 MJ·day^−1^ (2921 ± 506 kcal·day^−1^) however, 35% ± 5% of mean energy intake comprised of secondary items, which can be classified as snacking (4.28 ± 1.06 MJ·day^−1^, 1022 ± 253 kcal·day^−1^). This finding is supported by previous studies that identified contribution of snacks to total energy intake in high-level athletes range from 17%–22% [46] and 30%–37% [47]. Analysis of the self-reported energy intake identified that a number of secondary items were missed which equated to 0.33 MJ·day^−1^ (79 kcal·day^−1^). This finding clarifies 90% of the under-reporting error (−0.37 MJ·day^−1^ or −88 kcal·day^−1^), with the remaining 10% (0.04MJ·day^−1^ or 9 kcal·day^−1^) attributed to inaccuracy of weighing food items. This is an important consideration to acknowledge that actual reporting accuracy was not the issue; moreover it was the ability of the participant to record the consistent snacking throughout the day.

The mean observed energy intake (12.23 ± 2.12 MJ·day^−1^ or 2921 ± 506 kcal·day^−1^) is slightly higher in comparison to the Estimated Average Requirements [48]. Recommendations are 10.8 MJ·d^−1^ (2580 kcal·day^−1^) and 11.7 MJ·d^−1^ (2795 kcal·day^−1^) respectively for active male 13- and 14-years-old, based on Physical Activity Levels (PALs) of 1.85 [48]. It is important to recognize that although the recommendations [48] take in to consideration physical activity and growth, the increased physical activity levels experienced on a daily basis by the current study sample are considerably higher [16]. When comparing the results to free-living studies investigating nutritional intake of adolescent high-level soccer players, researchers have all reported sub-optimal energy intakes based on estimated energy expenditure [16,17,29], even when intake is in excess of mean observed energy intake in the current study. Energy intake findings of 14.3 ± 0.8 MJ·day^−1^ (3416 ± 191 kcal·day^−1^) [29]; 12.6 MJ·day^−1^ (3010 kcal·day^−1^) [17]; 11.9 ± 0.7 MJ·day^−1^ (2842 ± 167 kcal·day^−1^) [16] have all been identified, which demonstrate the current study values represent habitual energy intake in this population. The macronutrient breakdown of mean observed energy intake equated to carbohydrates (59% ± 3%), proteins (15% ± 3%) and fats (26% ± 3%). This finding is also in direct support of previous research with male adolescent soccer players as Russell and Pennock [16] identified macronutrient contributions to total energy intake as 56% ± 1%, 16% ± 1% and 31% ± 1% for carbohydrates, proteins and fats, respectively. Therefore, energy intake values of the present study are similar to that of habitual, free-living energy intake studies within a similar population [16], supporting the study’s ecologically valid design.

Rumbold *et al.* [26] recommended that studies assessing energy balance or devising exercise interventions, which require recording of energy intake should endeavor to establish the accuracy of the energy intake method, specific to the sample population. Whilst acknowledging the combined method requires high participant and researcher compliance, which may demand a relatively high time cost; the current study provides a benchmark for researchers and practitioners to use the combined method of energy intake when collecting such data within adolescent high-level soccer players in free-living environments. It is acknowledged that the two methods report a significant difference; however the magnitude of the difference is still considerably lower than previously published methods with male adolescents [6,10,11,18] and a suitable adjustment to self-report estimates of energy intake can be confidently applied given the narrowness of both the estimate of the bias score and of the random error between methods. Further research is required to utilize this combined method to assess energy balance in male adolescent high-level soccer players in a free-living environment over longer time scales. This will help to inform nutritional interventions to support the training and physical development of this population.

## 5. Conclusions

In conclusion, the combined method of self-report weighed food diary and 24-h recall demonstrated a low random error between methods and although a statistically significant under-reporting was observed, the magnitude of this bias was small. Applying an appropriate adjustment for under-reporting to the combined method could provide a valid alternative to current energy intake collection methods, providing both researchers and practitioners with a valuable tool to quantify energy intake in male adolescent high-level soccer players, in a free-living environment.
